# HOMLC-Hyperparameter Optimization for Multi-Label Classification of Intrusion Detection Data for Internet of Things Network

**DOI:** 10.3390/s23198333

**Published:** 2023-10-09

**Authors:** Ankita Sharma, Shalli Rani, Dipak Kumar Sah, Zahid Khan, Wadii Boulila

**Affiliations:** 1 Chitkara University Institute of Engineering and Technology, Chitkara University, Rajpura 140401, Punjab, India; ankitaanand2719@gmail.com; 2 Department of Computer Engineering and Applications, GLA University, Mathura 281406, Uttar Pradesh, India; 3 Robotics and Internet-of-Things Laboratory, Prince Sultan University, Riyadh 12435, Saudi Arabia; 4 RIADI Laboratory, National School of Computer Sciences, University of Manouba, Manouba 2010, Tunisia

**Keywords:** deep learning, IoT, security, low-rank representation, traffic data, support vector machines, convolutional neural network, multilayer perceptron

## Abstract

The comparison of low-rank-based learning models for multi-label categorization of attacks for intrusion detection datasets is presented in this work. In particular, we investigate the performance of three low-rank-based machine learning (LR-SVM) and deep learning models (LR-CNN), (LR-CNN-MLP) for classifying intrusion detection data: Low Rank Representation (LRR) and Non-negative Low Rank Representation (NLR). We also look into how these models’ performance is affected by hyperparameter tweaking by using Guassian Bayes Optimization. The tests has been run on merging two intrusion detection datasets that are available to the public such as BoT-IoT and UNSW- NB15 and assess the models’ performance in terms of key evaluation criteria, including precision, recall, F1 score, and accuracy. Nevertheless, all three models perform noticeably better after hyperparameter modification. The selection of low-rank-based learning models and the significance of the hyperparameter tuning log for multi-label classification of intrusion detection data have been discussed in this work. A hybrid security dataset is used with low rank factorization in addition to SVM, CNN and CNN-MLP. The desired multilabel results have been obtained by considering binary and multi-class attack classification as well. Low rank CNN-MLP achieved suitable results in multilabel classification of attacks. Also, a Gaussian-based Bayesian optimization algorithm is used with CNN-MLP for hyperparametric tuning and the desired results have been achieved using c and γ for SVM and α and β for CNN and CNN-MLP on a hybrid dataset. The results show the label UDP is shared among analysis, DoS and shellcode. The accuracy of classifying UDP among three classes is 98.54%.

## 1. Introduction

Differentiating between normal attacks and malicious attacks and classification of classes (known as multiclass classification) of attacks is undoubtedly existing tasks. However, in the scenario the problem is when many kinds of attacks occur simultaneously on a shared label known as multilabel classification, what would be the implications? The following work discusses the occurrence of multilabel classification keeping in mind the impact of both binary and multiclass classification. Machine learning and deep learning classifiers have been used for multi-label classification classifying more than one class of attacks on a common label [1,2,3,4]. Due to the fact that attacks can have a wide variety of characteristics, including type, severity, and target, it is necessary to develop efficient models in multi-label classification for attack classification. In addition, an attack may have various objectives, such as the theft of confidential information, the impairment of system performance, or the falsification of system output [5]. To assure the security and dependability of machine learning systems, attacks must be classified [6]. By identifying and categorizing attacks, security specialists and system administrators are able to mitigate their impact and prevent new ones from occurring. The classification of assaults, on the other hand, is a difficult endeavor that requires the application of cutting-edge machine learning technologies [7]. Effective models for multi-label attack classification [8] must take into account large and complex label spaces as well as the high dimensionality and variability of the input data. These models must also be resistant to adversarial attacks and adaptable enough to accommodate new attack techniques. The security and dependability of machine learning systems in a variety of industries, such as banking, healthcare, and critical infrastructure, are reliant on the development of efficient multi-label attack classification models [9]. Support Vector Machines (SVM) and Convolutional Neural Networks (CNN) are two prominent machine learning models that can be used in conjunction with low-rank factorization methods for the multi-label classification of assaults [10,11]. SVM is a supervised learning method that can complete classification tasks requiring both binary and multiple labels. SVM operates by locating a hyperplane that divides the data points into distinct classes and maximizing the distance between the hyperplane and the closest data points. SVM can be used to learn decision boundaries that separate multiple attack categories in multi-label classification. Using low-rank factorization techniques to reduce the dimensionality of the input data increases the efficacy of the SVM algorithm [12]. SVM may not be optimal for datasets with multiple dimensions or that are very large. SVM determines the hyperplane that optimizes the margin between classes, which can be a time-consuming process when dealing with a large number of data points or characteristics. In addition, SVM may not perform well when the data are highly imbalanced and some classifications are significantly more abundant than others. Consequently, deep learning approaches emerge [13]. CNN uses convolutional filters to extract features from data inputs. Using these characteristics, the provided data are then divided into numerous categories. CNN can be used to acquire characteristics from the input data and classify an assault into multiple categories when conducting multi-label classification of assaults. The effectiveness of the CNN model can be improved by employing low-rank factorization techniques to reduce the number of parameters [14]. The limitation of CNN is that it may require a large amount of training data to learn the relevant features for the classification task. CNN relies on the ability to extract meaningful features from the input data, and this process can be challenging when the data are complex or noisy [15]. Additionally, CNN may not be suitable for all types of input data, and may require some preprocessing or feature engineering to extract the relevant features. Low-rank factorization techniques also have some limitations. These techniques may not be suitable for all types of data and may require some assumptions about the underlying structure of the data. Overall, while SVM and CNN with low-rank factorization can be effective for multi-label classification of attacks, it is important to consider their limitations and choose the appropriate model based on the characteristics of the data and the classification task. Low-rank factorization is a technique for reducing the dimensionality of input data and improving the efficacy of machine learning models. Using low-rank factorization, the input matrix is partitioned into two or more low-rank matrices that represent the data’s essential characteristics. Specifically, high-dimensional data such as images or time series can benefit from this method. In general, low-rank factorization techniques combined with SVM or CNN can provide a viable solution for multi-label assault categorization, particularly in fields such as cybersecurity and network intrusion detection [16].

### Our Contributions

1. The use of a hybrid network intrusion dataset, i.e., merging of BoT-IoT and UNSW NB-15 dataset.
2. The performance of low-rank optimised SVM by determining the hyperplane efficiently for predicting classifiable labels on reproduced observations.
3. The weight optimization method through a low rank matrix factorization process on deep learning classifiers (CNN and CNN-MLP) for improvising multilabel classification.
4. The use of Bayesian optimization in conjunction with low-rank factorization SVM, CNN and CNN-MLP models, enables efficient hyperparameter tuning and model optimization.

## 2. Related Work

In [3], feature pre-processing techniques, namely feature extraction and feature scaling, were used on the BoT-IoT dataset and SVM achieved 79% accuracy in multi-class classification. The Bot-IoT dataset was also used in the study [5], which employed the models k-nearest neighbor (KNN), multi-layer perceptron (MLP), and Naive Bayes (NB). The research produced extremely good results in terms of accuracy, precision and F1 score. In this study, an updated dataset and a variety of classical learning models were used. Unfortunately, none of the models in this study were tested across multiple classes. Using [8], the two datasets BoT-IoT and UNSW NB-15 were used. The results were obtained by using specific features of both the datasets. The various classical learning models such as kNN, DT, NB and SVM-RBF were used. The accuracy obtained by both DT and NB is 67% on the 29 selected features whereas SVM-RBF obtained an accuracy of about 32% on 14 selected features with hyperparameters used such as C = 10 and γ = 0.0001. In [17], a combined IDS was developed using the C5 classifier and the One Class SVM. This study used a Bot-IoT dataset [18] containing IoT internet traffic with multiple types of cyberattacks to find common instructions. The various learning classifiers were used in [13], where the performance of NB and MLP were best on BoT-IoT dataset. Parameters such as accuracy, precision, recall and f-measure were calculated. The pre-processing techniques, namely feature extraction and feature selection, were used. These are the results with supervised learning algorithms, but results with unsupervised ones still need to be calculated. The deep learning classifiers including CNN and MLP individually applied on the Bot-IoT dataset where accuracy had been found based on different numbers of epochs and batch sizes so that the prevention of attacks that happen on an IoT network is feasible [14]. By using the BoT-IoT dataset and applying pre-processing techniques on it such as data resolution, missing port numbers and resolving data imbalance, several machine learning algorithms were used in [16] and SVM achieved 79% accuracy in multiclass classification. For multiclass classification, machine learning classifier SVM in [19] achieved an accuracy of about 88.3%. The pre-processing techniques used were missing values, entropy discretization and normalization. The multiclass classification had been performed on two of the datasets, i.e., BoT-IoT and UNSW NB-15 individually. The data pre-processing techniques such as one-hot encoding and min-max normalization had been applied. The classifiers such as MLP, SVM and DT were used. The experiment resulted in accuracy of about 72% for MLP, 59% of SVM, and 64% for DT in case of UNSW NB-15 dataset. While performing multiclass with BoT-IoT the classifiers achieved accuracies of about 91%, 94% and 92%, respectively. The multi-layer perceptron approach [20] is used in a variety of datasets, including DARPA and CAIDA for DDoS attack detection, to compare the effectiveness of various machine learning techniques. The usage of all ML algorithms being optimized reduces the rates of misclassification, which is the main disadvantage. With BoT-IoT, a novel method utilizing multi-layer perceptrons is used to assess accuracy as well as other hyperparameters [21]. Convolutional networks were utilized on UNSW-NB15 in [22], and accuracy metrics were acquired. Only specific attacks were classified, and the technique used was min-max formulation mixed with deep learning. The accuracy, detection rate, and false alarm rate performance measures for the deep learning CNN classifier that was deployed on the BoT-IoT yielded results only for the binary and multiclass classification of attacks, not for the multilabel classification [23]. The use of CNN in [10] for identification of attacks using the BoT-IoT dataset achieved an accuracy of about 89%. It is anticipated that this algorithm will be included into the NIDS so that it can be utilized real-time to counterattack threats. Also, there exist some of the different techniques used for low rank experimentation as shown in Table 1.

## 3. Methodology

### 3.1. Dataset Details

**Figure 1:**
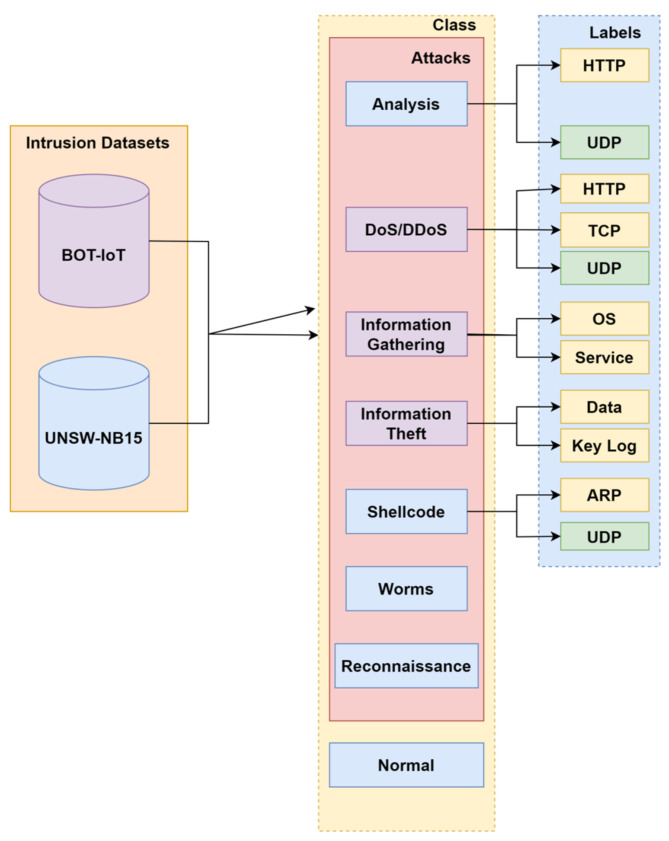
Integration of two datasets.

**Figure 2:**
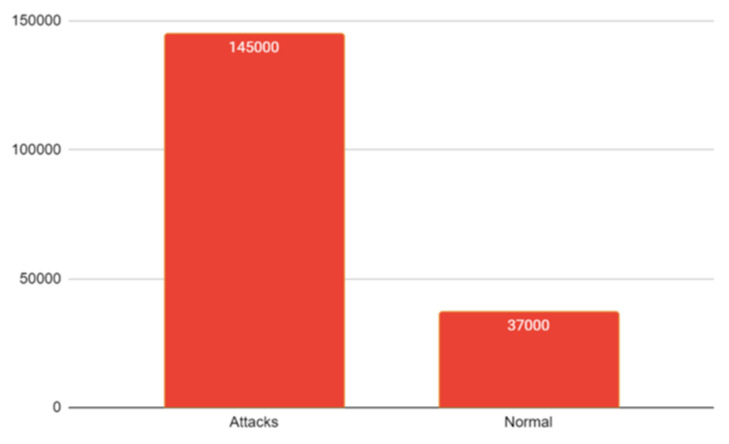
No. of classes having Normal and Attack.

**Figure 3:**
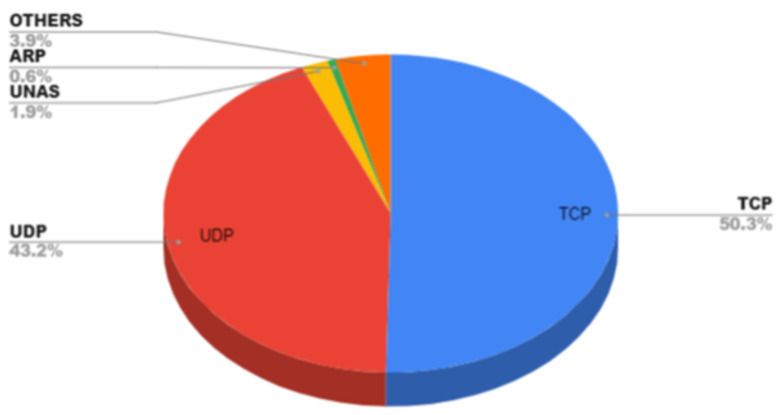
Types of protocols.

There exist many intrusion datasets such as CICIDS, DARPA, NSL-KDD, KDD CUP99, UNSW NB 15, BOT-IOT, etc. By combining the two datasets, BoT-IoT and UNSW NB-15, an effort has been made to find a workable solution for the classification of attacks when multiple labels are present while also considering the influence on binary and multiclass classification. The features in both the datasets are of datatypes such as categorical, numeric and nominal [35]. At the time of analysis of the dataset to check whether the dataset is fit for the experiment or not, an exploratory data analysis for visualizing the data has been conducted where the total number of entries, rows and columns with missing values, redundant values, nominal, categorical and numeric datatypes, filling of the missing values, removal of attributes, data type conversion, correlation, univariate, bivariate and multivariate analysis, k cross fold stratified technique for the validation process, etc., have been checked [18]. The first step of preprocessing the data while concatenating the two intrusion datasets on the basis of column ‘label’. All variables from both the data sets are included. Variables whose values are present in one data set but not in the other are marked as missing values. Cleaning of data is the initial requirement where missing data and redundant data are the major problems [23]. The redundancy of data should be removed because at the time of applying the k-fold cross validation strategy there will be less chances for accurate and precise results. One-hot encoding is used to convert categorical text into numeric datatype. Correlation function is used to check the relationship among the features [20]. The next step is the implementation of discretization on the combined dataset, the collection of cut points that will segment a quantitative attributes range of values into a limited number of intervals with good class coherence. The following dataset uses a correlation technique to check the relationship among different features. The maximum value range varies greatly. A normalized processing method is used to uniformly and linearly map the value range of each feature within the [0, 1] intervals, which simplifies arithmetic processing and the elimination of dimensions [36]. The integrated dataset is shown in Figure 1 also having normal and attack type is shown in Figure 2 with total instances of 187,000. The Figure 3 is showing the total number of protocols in the integrated dataset.

### 3.2. Data Pre-Processing and Mixing

The first stage of data pre-processing is the integration of two incursion datasets based on the ‘label’ column. The hybrid dataset refers to the collective observations derived from the integration of several data sets. All variables from both datasets are included [21]. Missing values are assigned to variables that have values present in one dataset but not in another. Within this hybrid dataset, there are instances that have missing values. Data cleaning, sometimes referred to as data imputation, is a crucial process that involves addressing the issue of missing and duplicate data [14]. In order to obtain exact and reliable results, the missing values may be handled by either leaving them blank, filling them in manually, replacing them with the attribute mean, or substituting them with the most probable value. By disregarding the tuple or column that has missing data, there is a significant likelihood of obtaining inaccurate outcomes and also forfeiting essential information [16]. One limitation of imputing missing values with the mean is that this method is only applicable to numeric data types, and does not account for categorical or ordinal variables included in the dataset [37]. The elimination of data redundancy is necessary in order to enhance the accuracy and precision of findings while using the k-fold cross-validation approach. One-hot encoding is used as a means of converting categorical textual data into a numeric data format [36]. The correlation function is used to assess the association between the characteristics [19].

### 3.3. Discretization

In order to apply discretization to the combined dataset, a set of cut points will be used to divide the range of values of a quantitative attribute into a finite number of intervals that exhibit strong class coherence [38]. Data discretization is a technique used to replace numeric attribute values with intervals. The class-attribute contingency coefficient, like a typical classification approach, may lack the ability to accurately calculate the correct discretization intervals when applied to multi-label data [22,24]. The concept of class-attribute interdependency refers to the relationship between a class and its associated attributes within a given context. This inter-maximization is a supervised discretization approach that incorporates knowledge of class information. Its objective is to automatically determine the optimal number of discrete intervals and cut points, taking into account the relationship between the class and attribute value maximization [39]. This methodology is used to achieve the aforementioned objective of identifying the optimal discretization technique. One additional objective is to decrease the quantity of intervals while maintaining interdependence among class features. Data discretization is a technique used to replace numeric attribute values with intervals [17].

### 3.4. Normalization

The range of maximum values varies widely. In order to facilitate arithmetic processing and elimination of dimensions, a normalized processing method is adopted to uniformly and linearly map the value range of each feature within the [0, 1] intervals [23]. After attaining numerical values in the dataset with a attributes and b labels, min(a) is the minimum value of attribute and max(a) is the maximum value of attribute where the difference between these max and min values have a large scope, and as a result min-max normalization technique can be applied [18].

### 3.5. Low Rank Factorization

In the following work, non-negative matrix factorization (NMF) has been used to reduce the dimensionality of the dataset, extract the most important characteristics, and preserve the interpretability of the data. The original dataset is divided into two non-negative matrices by NMF, each of which holds the related feature weights and latent features [9]. The created latent characteristics can then be used as inputs to machine learning models to improve their effectiveness in detecting abnormalities and identifying attack patterns.

In order to implement the non-negative matrix factorization on the high-dimensional and sparse dataset, the matrix A can be defined as follows:

(1) $$W\approx MN^{T}$$

where *M* is an a × b matrix with a instances and b features, *N* is an a × r matrix containing a instances and r latent features, and $A^{T}$ is an b × r matrix containing r latent features and b features. The goal of low-rank matrix factorization is to find the matrices *N* and *M* that minimize the reconstruction error between *M* and $MN^{T}$, subject to the low-rank constraints. To extract important features and to reduce data’s high dimensionality, a low rank matrix $MN^{T}$ has been produced.

(2) $$rank(MN^{T})\leq r$$

Finding the ideal choices for *M* and *N* that minimize the reconstruction error and meet the low-rank requirements is the aim of the optimization issue. Numerous methods, such as gradient descent or alternating least squares, can be used to solve this problem. The ideal values for *M* and *N* have been determined, the latent features included in *N* can be employed as inputs to deep learning models to improve their efficiency in identifying attack patterns and detecting anomalies [12]. A low-rank coefficient matrix seeks out one, recognizes, and accurately represents the data sets’ structure. Equation (3) demonstrates the definition of the sparsecoding-like model for clean data.

(3) $$min_{Z}{\left\|Z\right\|}_{*}s.t.X=XZ$$

The given solution Q for above is shown in Equation (4):

(4) $$Z=N_{X}N_{X}^{T}$$

where $N_{X}$ are column vectors, *X* are singular vectors. X=XZ is considered as the least square solution. The entries E in the column vectors and singular vectors $N_{X}$ $N_{X}^{T}$ can intuitively be zero. Low rank matrix factorization functions better in finding the entire feature space design and removing outliers from the data at the same time. It defines a nuclear norm minimization to handle distorted high-dimensional data as shown in Equation (5):

(5) $$min_{Z},E\left\|Z\right\|+\lambda {\left\|E\right\|}_{2},1^{\prime }s.t.X=MZ+E$$

### 3.6. LR-SVM

**Figure 4:**
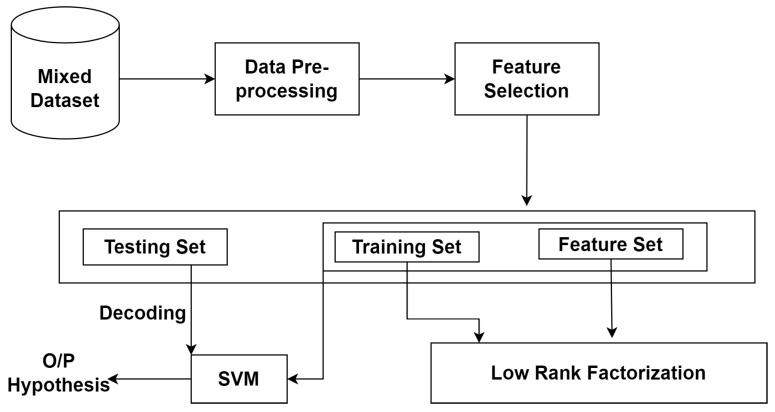
Proposed methodology LR-SVM.

Figure 4 shows a hybrid dataset that has been used for solving the multilabel classification for the recognition of shared label among different classes. The discussion and working of baseline model and LR-SVM is in [40]. The pre-processing techniques has been used as the dataset after merging contains redundancies and inconsistencies. The first step is data cleaning to fill missing values with that of the mean values in the column. The data discretization has been used to convert the nominal and categorical values in that of the discrete values by entropy binning method so that maximum boundaries can be induced on the sorted list [19].

The removal of outliers or repetitive values and the use of Z-score normalization have come into the picture. After applying the three pre-processing techniques using correlation where high dependabilities among features are selected based on that, highly correlated features are selected. The dataset has been divided into 80–20 rule for training and testing. A decision matrix has been obtained by 80% of training of the highly correlated features by using a k-cross-fold stratified validation comprising alternatives and criteria [38]. The process of LR-SVM is described in [40]. The decision matrix can be normalized by

(6) $$x_{i_{j}}=\frac{u_{ij}}{\sqrt{\sum_{k=1}^{p}}u_{k_{j}}^{2}}$$

i=1…p, j=1…q where $u_{ij}$ is the score or threshold of alternative $A_{i}$ and criteria $C_{j}$.

The evaluation of alternatives by the row values of features and evaluation of criteria on the basis of shared labels correspond to the classes of attacks. The present dataset with alternatives and criteria has not been representative to the complete dataset. To make the dataset representative, the use of weights has been introduced. The first method of assigning weights is based on “assumption”, which is a vague method. The second method is using the weights of the pre-trained weights of the baseline model, which is known as weighted mapping. The weighted normalized decision matrix can be calculated as

$$Z_{ij}=W_{i}Xx_{ij};j=1\ldots p,i=1\ldots q$$

Let $W_{i}$ = $\left[w_{1},w_{2}\ldots w_{q}\right]$ be the local criteria weight vector with the value $\sum_{i=1}^{n}W_{i}=1$.

A threshold has been selected, which is obtained as the average value of alternatives and criteria. Based on this threshold value, if the value obtained is less than the threshold value a Negative Ideal Solution is obtained, otherwise if the value obtained is more than or equal to the threshold value the positive ideal solution is obtained. Based on this moving towards negative ideal solution, the results are non-achievable. The desired results are achieved by moving towards the positive ideal solution. For positive $\left(I^{+}\right)$ and negative $\left(I^{-}\right)$ ideal solutions

(7) $$\left(I^{+}\right)=z_{1}^{+}\ldots z_{q}^{+}=\left(max_{i}Z_{ij}|j\in J\right)\left(min_{i}Z_{ij}|j\in J^{\prime }\right)$$

(8) $$\left(I^{-}\right)=z_{1}^{-}\ldots z_{q}^{-}=\left(min_{i}Z_{ij}|j\in J\right)\left(max_{i}Z_{ij}|j\in J^{\prime }\right)$$

where *J* is the benefit criteria and $J^{\prime }$ is the cost criteria.

The point near the threshold value is the relative closeness and low-ranking towards positive ideal solution has been accomplished. Relative Closeness (RC) to the ideal solution is

(9) $$RC_{i}=\frac{N_{i}^{-}}{N_{i}^{+}-N_{i}^{-}}$$

Ranking as per $RC_{i}$ = (i = 1, 2 …n) where $RC_{i}$ = 1 indicates the highest rank and $RC_{i}$ = 0 indicates the lowest rank.

The results obtained were fed to SVM, where the use of radial basis function kernel and hyperparameters “C” and “gamma” has been optimized using different values and the desired results have been obtained on epochs and the learning rate.

### 3.7. LR-CNN-MLP

**Figure 5:**
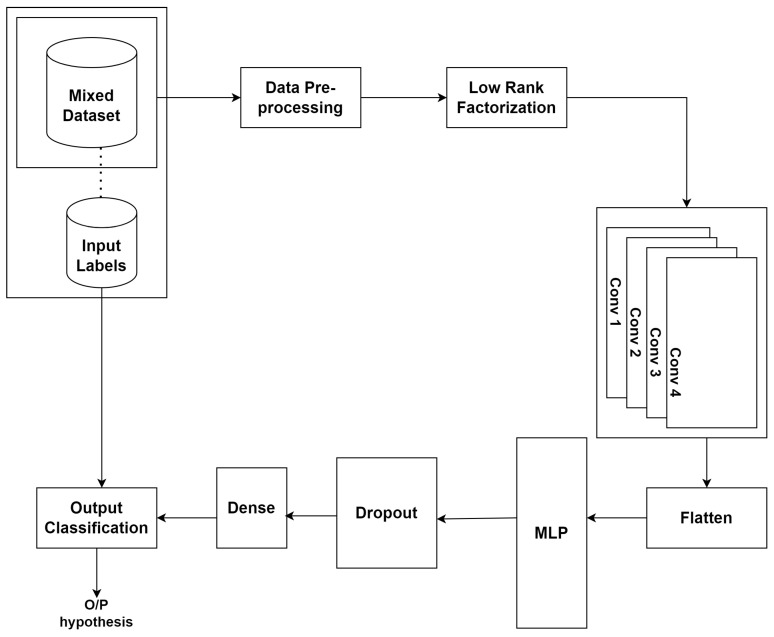
Proposed methodology LR-CNN-MLP.

The proposed method shown in Figure 5 enhances the performance of multi-label classification on CSV datasets by combining the positive attributes of CNNs and low-rank matrix factorization. The matrix factorization layer and the convolutional neural network layer compose the MF-CNN architecture. The matrix factorization layer employs NMF or singular value decomposition (SVD) methods to divide the initial input matrix P into two low-rank matrices, A and B. The latent properties of the input data are represented by the decomposed matrices A and B, which can be utilized to train the CNN layer. The CNN layer uses the well-known convolutional and pooling algorithms to extract high-level properties from the input data after receiving the deconstructed matrices A and B. The acquired features are then processed through a fully connected layer with softmax activation to obtain the final multi-label classification results. The attack dataset, i.e., the hybrid dataset has been used and fed as input. The same three preprocessing techniques have been implemented. After loading the dataset, the dataset has been split into a training set, validation set and testing set. The low rank factorization has been performed on the training dataset to obtain low dimensional representation. The hyperparameter, i.e., rank “r” has been defined. The decision training matrices with random non-negative values have been initialized. The CNN model has been built using multiple convolutional and pooling layers. The hyperparameters such as number of filters, filter size, activation function and pooling size have been defined. The low dimensional representation obtained above has been used as an input to CNN model. The relevant data features have been extracted by applying convolutionals, activation function and pooling operations.

(10) h=relu(W∗R+b)

where *W* is the set of convolutional filters of size (F × K), *b* is the bias term of size F, relu is the rectified linear unit activation function, and *h* is the output feature maps of size (F × S).

(11) p=maxpool(h)

where maxpool is the max pooling operation and *p* is the output pooled feature maps of size (F × R), R is the reduced spatial dimension.

The output of the final pooling layer has been flattened. The MLP model with multiple fully connected layers has been built. The hyperparameters such as number of neurons and activation function have been defined.

(12) $$Z_{1}=f\left(W_{1}*X+b_{1}\right)$$

where $W_{1}$ is the weight matrix of the first hidden layer, $b_{1}$ is the bias vector, f is the activation function, and $Z_{1}$ is the output of the first hidden layer.

The flattened output from the CNN and low dimensional representation has been concatenated and to obtain hidden representations linear transformations and activation function have been applied. To prevent overfitting, dropout regularization has been applied to hidden representations. To obtain probability estimates for each label linear transformation, sigmoid functions have been applied to the output of the final layer. After this, the model has been trained using training and validation sets. The binary cross entropy loss function has been defined, and we initialize the model weights with random values. To optimize the model weights, stochastic gradient descent with backpropagation has been used. Using the validation set, the hyperparameters have been tuned. Now the model has been tested using the testing set. The trained model has been used to make predictions on the testing set. The performance metrics such as accuracy, precision and recall have been evaluated. The same steps have been repeated for multiple runs with different random initializations of the low-rank matrix factorization and model weights to obtain an ensemble of models. We output the probability estimates for each label in the multi-label classification problem as the average of the probability estimates obtained from the ensemble of models.

### 3.8. Bayesian Optimization

The traditional methods of manually running the models can be inefficient, sensitive to noise, and difficult to use in cases where the objective function is not well-defined. Likewise, grid search involves evaluating the objective function at every point in a pre-defined grid, which can be very time-consuming, especially if the objective function is expensive to evaluate. On the other hand, there exists a technique called random search, which involves evaluating the objective function at a random subset of points, which can also be time-consuming and may not yield accurate results if the objective function is noisy. However, traditional optimization techniques require the user to specify a search space, which can be difficult to accomplish in cases where the objective function is not well-understood. This is because the user must know the possible values that the hyperparameters can take on in order to specify a search space [11]. Here, Bayesian optimization uses a probabilistic model to estimate the best next point to evaluate, which can help to avoid wasting time and resources on evaluating points that are unlikely to be optimal. Moreover, the number of evaluations required to discover the optimal solution is also reduced. Thus, it can be concluded as a sequential model-based optimization technique that uses surrogate models and acquisition functions to direct the search for the best solution in the most efficient way possible. It is an effective method for optimizing hyperparameters. Algorithm 1 discusses the optimal selection of hyperparameter using Gaussian-based Bayesian parameter optimization for multi-label classification.

The alpha (α) and beta (β) hyperparameters in Bayesian optimization algorithms primarily relate to the prior distribution hyperparameters used in Bayesian inference. The form and features of the prior distribution over the unknown function being modeled are influenced by these hyperparameters. The alpha hyperparameter, denoted as α, is associated with the prior mean function of the Gaussian process surrogate model used in Bayesian optimization. It computes the overall mean value of the function under consideration. A greater alpha value corresponds to a higher prior mean function, meaning that the objective function will have a higher expected value. The beta hyperparameter, denoted as β, is associated with the prior covariance function (kernel) of the Gaussian process surrogate model. It controls the smoothness or roughness of the function being modeled. A lower value of beta indicates a smoother function, whereas a higher value of beta results in a more rough or oscillatory function.
**Algorithm 1.** Optimal selection of hyperparameter using Gaussian-based Bayesian parameter optimization for multi-label classification.**Input:** Attack dataset in CSV format (dataset)**Output:** Probability estimates for each label in the multi-label classification problem**Step 1:** Load the dataset and split it into training $X_{train}$ and testing sets $X_{test}$.**Step 2:** Perform data pre-processing on $X_{train}$.**Step 3:** Define the rank r, no. of classes n, no. of labels k, iters, learning rate lr, epochs and optimizer as hyperparameters.**Step 4:** Initialize CNN-MLP model and train it on $X_{train}$ and target labels Y (n * k).spm ← model ($P_{score}$(conv($X_{train}$)))/$P_{hyp}$(conv($X_{train}$))**Step 4.1:** Apply the parameters to objective function$hyp^{*}$ = arg $min_{hyp\in k}$ f(k)**Step 4.2:** Update surrogate probabilistic model for new parameters.spm ← model ($P_{score}$(conv($X_{train}$)))/$P_{hyp}$(conv($X_{train}$))**Step 5:** Obtain weights ‘W’ of last fully connected layer.**Step 6:** Perform low-rank matrix factorization on ‘W’.W = $M_{train}$$N_{train}$ Twhere $M_{train}$ = h*r$N_{train}$ = r*k**Step 7:** Initialize r with random values.**Step 8:** Define a function ‘$update_{r}$$(M_{train}$, $N_{train}$, lr)’ at last fully connected layer.**Step 8.1:** Obtain the low-rank matrix until convergence:$M_{train}$ = $M_{train}$ * ($X_{train}$ * $N_{train}$)/($M_{train}$ * $N_{train}.T$ * $N_{train}$)$N_{train}$ = $N_{train}$ * ($X_{train}.T$ * $M_{train}$)/($N_{train}$ * $M_{train}.T$ * $M_{train}$)**Step 8.2:** Compute the residualsE=W-$M_{train}$$N_{train}$ T**Step 8.3:** Compute the gradient of ‘$M_{train}$’ ‘$N_{train}$’ w.r.tgrad $M_{train}$ =−2E −$N_{train}$grad $N_{train}$= −2E T $M_{train}$**Step 8.4:** Update $M_{train}$ and $N_{train}$ using learning rate as$M_{train}$ = $M_{train}$−(lr* grad $M_{train}$)$N_{train}$ = $N_{train}$−(lr* grad $N_{train}$)**Step 8.5:** Update the weights as last fully connected layers.W = $M_{train}$$N_{train}$ T**Step 9:** Replace the weights of last fully connected layer in CNN-MLP with updated weights$M_{train}$$N_{train}$.**Step 10:** Retrain the CNN-MLP model on $M_{train}$ and target labels Y using fully connected layer.**Step 11:** Repeat Steps 5–9 for ’iters’ iterations**Step 12:** Probability estimates for each label in the multi-label classification problem as the average of the probability estimates obtained from the ensemble of models.

## 4. Results and Discussion

The following section discusses the results based on low rank factorization with SVM, CNN and CNN-MLP.

### 4.1. Parameter Tuning for Proposed SVM-Based Attack-Type Label Classification

Due to the class representation being imbalanced, the weighted classes parameter has been used. This allows the disparity of the classes to be rectified, the results of which are shown in Table 2, where the results are based on the different number of epochs and learning rates. The role of learning rate in optimization is used to determine the step size at each loop with a minimum of a loss function. The number of epochs are 200 with a learning rate of 0.015 that achieved the best results with 87.26% accuracy.

### 4.2. Parameter Tuning for Proposed CNN and CNN-MLP Based Attack-Type Label Classification

**Figure 6:**
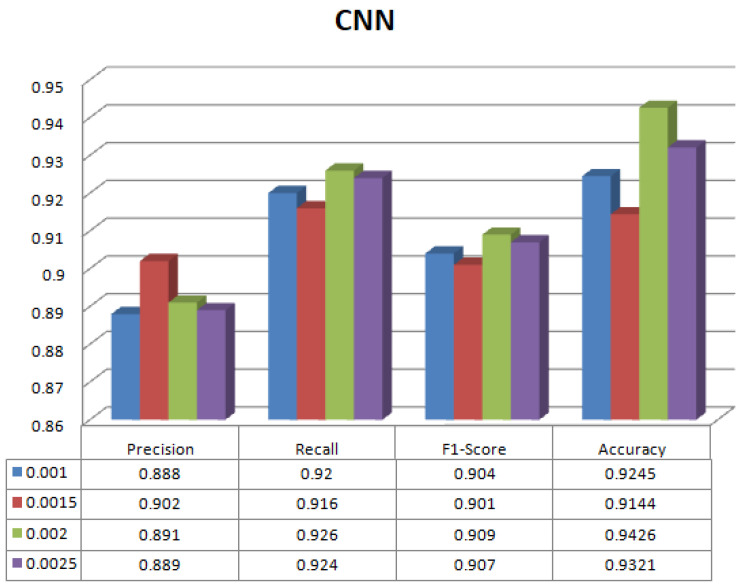
Parameters for LR-CNN on different learning rates.

**Figure 7:**
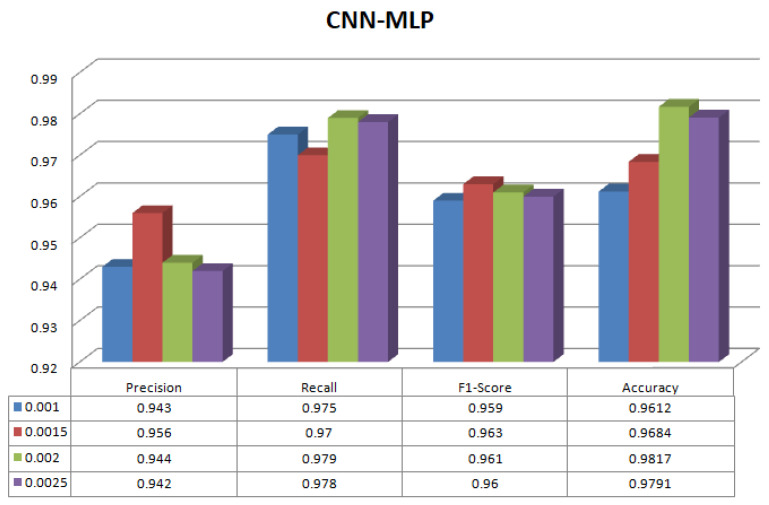
Parameters for LR-CNN-MLP on different learning rates.

**Figure 8:**
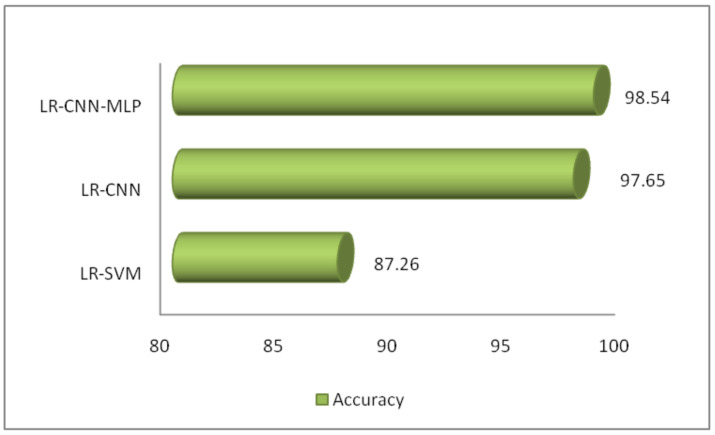
ML/DL classifiers with accuracy.

Table 3 shows the results of the proposed methodology (LR-CNN) on multilabel classification. The experiment has been conducted on different learning rates. The best result is on learning rate 0.0020 where precision is 0.891, recall is 0.926, f1-score is 0.909 with highest accuracy of 94.26%. Figure 6 shows different parameters for LR-CNN on different learning rates. Table 4 shows the results of proposed methodology (LR-CNN-MLP) on multilabel classification. Again, the experiment has been conducted on different learning rates. The best result is on learning rate 0.0020 where precision is 0.944, recall is 0.979, f1-score is 0.961 with highest accuracy of 98.17%. Figure 7 shows different parameters for LR-CNN-MLP on different learning rates. Both LR-CNN and LR-CNN-MLP give the best results on learning rate of 0.0020. Therefore, no impact of different learning values has been observed. Figure 8 shows the ML and DL classifiers along with their corresponding accuracies.

### 4.3. Parameter Tuning for Guassian Based Bayesian Optimization Algorithm

**Figure 9:**
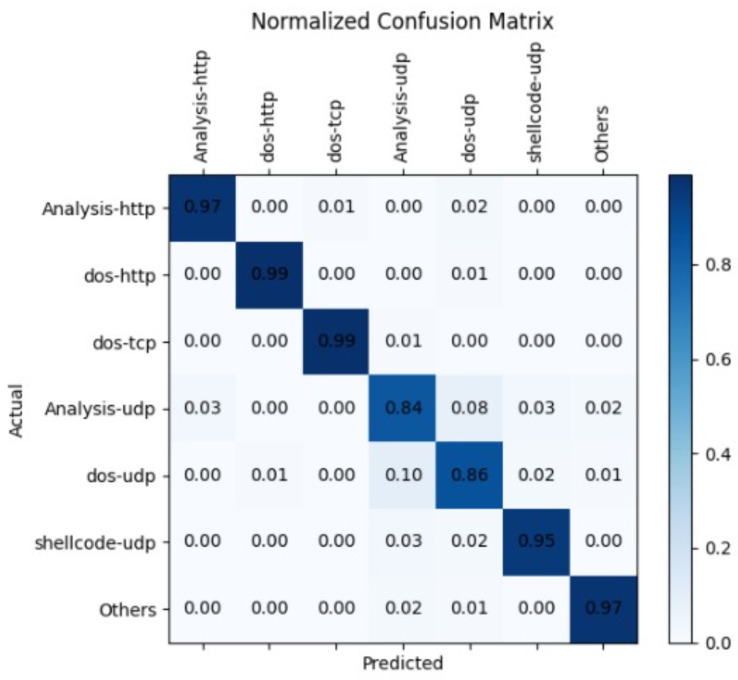
Confusion matrix corresponding to the Bayesian optimization of the proposed model of LR-CNN-MLP.

Table 5 shows the results on multilabel classification using Bayesian optimization. The accuracy 87.26% is the same and without any change in parameters c and γ, lr = 0.0020, epochs = 150 and optimizer SGD using low-rank SVM. The best two readings selected using low-rank CNN having parameters α = 1, β = 0.5, lr = 0.001, epochs = 100, layers = 2 and ADAM optimizer achieved accuracy of 96.36%. The parameters α = 1, β = 0.5, lr = 0.002, epochs = 200, layers = 2 and SGD optimizer achieved accuracy of 97.65%. The best three readings selected using low-rank CNN-MLP having parameters α = 1, β = 0.5, lr = 0.001, epochs = 100, layers = 2 and ADAM optimizer achieved accuracy of 98.89%. The parameters α = 1, β = 0.5, lr = 0.0015, epochs = 125, layers = 2 and SGD optimizer achieved accuracy of 99.20%. The parameters α = 1, β = 0.5, lr = 0.0020, epochs = 125, layers = 2 and RMSprop optimizer achieved accuracy of 98.54%. The label UDP is shared among analysis, dos and shellcode. The accuracy of classifying UDP among three classes is 98.54%, as it successfully dealt with the framed objectives, properties of datasets and other levels of coarseness. All these factors together became important for the prediction of labels individually showing insight knowledge using a confusion matrix as shown in Figure 9.

### 4.4. Limitation

The following work is limited to the datasets having a shared label and not being applicable to the dataset where common labels will be absent. Also, the normalized confusion matrix has been able to identify the common label UDP among analysis, shellcode and DoS classes of attacks, but still UDP along analysis and dos are still a point of concern due to less difference among the predicted and actual classes of attacks.

## 5. Comparative Analysis

The UNSW-NB 15 dataset is used in [41], where convolutional neural network and multi layer perceptron deep learning classifiers had been used individually for attack classification in which the results for root mean square error were good but with a drawback of non-customization as shown in Table 6.

In [41], a convolutional network was used on UNSW-NB15 and an accuracy metric was obtained, and the technique used was min-max formulation combined with deep learning where only specific attacks were classified. The deep learning CNN classifier implemented on BoT-IoT and accuracy, detection rate and false alarm rate performance metrics were obtained and achieved results in binary and multiclass classification of attacks only, but not on multilabel classification [43]. In [44], a binary and multiclass convolutional network is implemented on UNSW-NB15 where four metrics such as accuracy, precision, recall and f-measure were obtained. A Skip connection methodology into CNN did not performed well on the specific dataset. A multilayer perceptron [45] classifier was applied on BoT-IoT where precision and f1-score were obtained. It helps to monitor traffic flow in connected host, but only worked in binary classification. In [46], a convolutional deep network was implemented on BoT-IoT dataset where parameters such as accuracy, precision and training time parameters were used to achieve the desired results and CNN performed well in comparison to linear regression and decision trees, but computational and memory utilization can be still improved by using optimized generalized techniques.

## 6. Conclusions

As security plays an important role in every field, therefore a secure model has been developed using machine learning and a hybrid deep learning model. A hybrid security dataset has been taken and used with low rank factorization in addition to SVM, CNN and CNN-MLP. The desired multilabel results have been obtained by considering binary and multiclass attack classification as well. Low rank CNN-MLP achieved suitable results in multilabel classification of attacks. Also, a Gaussian-based Bayesian optimization algorithm is used with CNN-MLP for hyperparametric tuning and the desired results have been achieved using c and γ for SVM and α and β for CNN and CNN-MLP on a hybrid dataset, i.e., merging of UNSW NB-15 and BoT-IoT datasets.

## 7. Future Scope

The potential for creating and applying hybrid deep learning models for classifying threats in the IoT domain is very promising in the future. As the IoT continues to expand and integrate into many sectors, ensuring strong security measures becomes more imperative. Hybrid deep learning models may provide a complete methodology for the detection and classification of threats by utilizing the advantages of several methodologies, hence improving accuracy and resilience. Here are some key aspects for the future scope.

Accuracy and Resilience: Hybrid models have the capability to integrate the strengths shown by several deep learning architectures, such as convolutional neural networks, recurrent neural networks, and transformers, with standard machine learning approaches. This amalgamation results in improved accuracy and resilience. The integration of this fusion technique has the potential to improve the accuracy and resilience in the identification and categorization of diverse attacks on IoT systems.

Transfer Learning: The use of transfer learning and pretraining may provide advantages in scenarios when there is a scarcity of labeled attack data. Transfer learning involves the fine-tuning of a pretrained model that has been trained on a task relevant to attack categorization. Hybrid models may use transfer learning to alter pre-trained models for the purpose of detecting IoT attacks.

Shortage of labeled data: The shortage of labeled data in IoT systems may arise from the infrequent occurrence and evolving nature of anomalies and assaults. This presents a challenge for semi-supervised and unsupervised learning approaches. Hybrid models have the capability to include unsupervised learning methodologies, such as autoencoders or generative adversarial networks (GANs), in order to effectively identify and classify previously unseen attack patterns, hence enabling the discovery of anomalies.

Ensemble methods: Ensemble methods refer to a class of machine learning techniques that combine several individual models to make predictions or decisions. These methods aim at ensemble approaches, which integrate the predictions of numerous models, have the potential to improve generalization and overall performance. Robust ensemble frameworks may be constructed by combining traditional machine learning classifiers or rule-based systems with hybrid deep learning models.

Real-time detection: Real-time detection is a crucial aspect in the context of IoT systems, as it serves to mitigate possible harm by promptly identifying and responding to attacks. This necessitates the implementation of attack detection mechanisms that operate in real-time or with minimal latency. Hybrid models have the potential to be tuned in order to achieve low-latency processing, hence facilitating the prompt identification and mitigation of attacks.

Continuous Learning and Adaptation: IoT ecosystems exhibit dynamic characteristics, and the patterns of attacks might undergo evolutionary changes over time. Hybrid models provide the capability to include methodologies for continuous learning, hence facilitating their ability to adjust to emerging attack types and uphold classification accuracy.

Privacy-preserving solutions: The data generated by the IoT sometimes include confidential and private information. Hybrid models have the capability to safeguard sensitive data during model training by using methodologies such as federated learning or differential privacy.

In summary, the potential for hybrid deep learning models to classify IoT threat types is significant. In order to enhance the security of IoT systems in light of emerging threats, it is imperative for researchers and practitioners to devise innovative methodologies that integrate the strengths of many methods, therefore yielding accurate, resilient, and flexible solutions.

## Figures and Tables

**Table 1:** Existing techniques with advantages and limitations.

Ref. No./Year	Technique	Data-Cleaning	Discretization	Normalization	Advantages	Limitations
[24]/ 2015	Inexact Augmented Lagrange Multiplier	X	X	X	For complex data LRSR with spatial clustering for better performance.	LRR and LRSR not shown much improvement for simple data. Occupying single subspace.
[25]/ 2016	Inexact Augmented Lagrange Multiplier	✓	X	X	Instead of occupying single subspace, occupies multi subspace. Removal of Outliers and Noise with no computational cost.	LRS coefficient matrix is missing.
[26]/ 2016	Linearized alternating direction method (LADM)	X	X	✓	LRS coeffient matrix is obtained using LADM.	No systematical way to estimate parameters Lambda 1 and Lambda 2.
[27]/ 2017	Alternating direction method (ADM)	✓	X	✓	Fixed Lambda = 2, noise free data with independent subspaces.	Aim to obtain an effective data representation matrix is still needed.
[28]/ 2019	Wilcoxon Signed Rank	X	X	X	Fast and Flexible model with non-linear behavior and representation of data matrix.	Low detection performance.
[29]/ 2019	SAW, TOP SIS, MCM	X	X	X	The collected samples’ low rankness in low-dimensional space is used to create an instructive graph that captures local information.	Capturing global information is still an issue.
[30]/ 2019	Low-Rank Representation	X	X	X	Both local and global info of the original samples can be well captured.	Insufficient creation of dictionary
[31]/ 2019	LRaSMD	X	X	✓	Proper dictionary is created using LR and SM.	Single distribution can be used to simulate both anomalies and noise, which separates weak anomalies and noise.
[32]/ 2020	Manhattan Distance LSMD-MoG	X	X	✓	Single distribution is replaced by MoG. Not only stable but also effective for hyperspectral AD.	Lambda and beta set to 0.1.
[33]/ 2020	LELRP-AD	X	X	X	Low rank property of DM is enhanced.	The rank value r and cardinality c taken to be specific.
[34]/ 2021	Manhattan Distance LSMD-MoG	X	X	✓	Finds all anomalies and shapes them clearly.	WSL can not be used without LRR.

**Table 2:** Multi-label classification results on proposed methodology (LR-SVM).

Epochs	Learning Rate	Accuracy
Epochs = 150	lr = 0.010	85.35%
	lr = 0.015	81.34%
	lr = 0.020	82.21%
	lr = 0.025	82.56%
**Epochs = 200**	lr = 0.010	85.45%
	**lr = 0.015**	**87.26%**
	lr = 0.020	85.65%
	lr = 0.025	86.69%
Epochs = 250	lr = 0.010	83.20%
	lr = 0.015	86.24%
	lr = 0.020	86.54%
	lr = 0.025	86.44%

**Table 3:** Multi-label classification results on proposed methodology (LR-CNN).

Learning Rate	Precision	Recall	F1-Score	Accuracy
lr1 = 0.0010	0.888	0.920	0.904	92.45
lr2 = 0.0015	0.902	0.916	0.901	91.44
**lr3 = 0.0020**	**0.891**	**0.926**	**0.909**	**94.26**
lr4 = 0.0025	0.889	0.924	0.907	93.21

**Table 4:** Multi-label classification results on proposed methodology (LR-CNN-MLP).

Learning Rate	Precision	Recall	F1-Score	Accuracy
lr1 = 0.0010	0.943	0.975	0.959	96.12
lr2 = 0.0015	0.956	0.97	0.963	96.84
**lr3 = 0.0020**	**0.944**	**0.979**	**0.961**	**98.17**
lr4 = 0.0025	0.942	0.978	0.960	97.91

**Table 5:** Observational performance evaluation on selection of parameters.

Model	Parameter	Accuracy
LR-SVM	c = 1.0, γ = 0.1, lr = 0.0020, epochs = 150, and optimizer = SGD	87.26%
LR-CNN	α = 1.0, β = 0.5, lr = 0.001, epochs = 100, layers = 2, and optimizer = Adam	96.36%
	α = 1.0, β = 0.5, lr = 0.002, epochs = 200, layers = 2, and optimizer = SGD	97.65%
LR-CNN-MLP	α = 1.0, β = 0.5, lr = 0.001, epochs = 100, layers = 2, and optimizer = Adam	98.89%
	α = 1.0, β = 0.5, lr = 0.0015, epochs = 125, layers = 2, and optimizer = SGD	99.20%
	α = 1.0, β = 0.5, lr = 0.0020, epochs = 125, layers = 2, and optimizer = RMSProp	98.54%

**Table 6:** Comparative analysis.

Ref. No./Year	Dataset	DL Classifier	Parameters	Findings	Limitations
[41] 2019	UNSW-NB15	CNN, MLP	Accuracy and F1-Score	Good performance in terms of RMSE.	Not easily customizable.
[42] 2020	UNSW-NB15	CNN	Accuracy	Analysis of min-max formulation with DL.	Specific attack types.
[43] 2020	Bot-IoT	CNN	Accuracy, Detection Rate, FAR	Results is binary and multiclass classification	No results for multilabel classification
[44] 2021	UNSW-NB15	BCNN MCNN	Accuracy, Precision, Recall, F-measure	Skip connection methodology into CNN	Not performed well on the specific dataset.
[45] 2021	Bot-IoT	MLP	Precision and F1-Score	Help to monitor traffic flow in connected host	Only worked in binary classification
[46] 2022	Bot-IoT	CNN	Accuracy, Precision and Training Time	CNN achieved best results as compared to LR and DT	Computational and memory utilization can be improved by using optimized generalized techniques.
Proposed Methodology	Hybrid dataset	LR-SVM	Accuracy	SVM has not been able to achieve results in multilabel classification of attack classes corresponding to one label	Due to high sparsity and high dimensionality of data in the integrated dataset
Proposed Methodology	Hybrid dataset	LR-CNN-MLP	Accuracy, Precision, Recall and F1-Score	Hybrid CNN-MLP achieved results in multilabel classification of attack classes corresponding to one label	

## Data Availability

Data are available publicly and given in references.
